# Tolerance and Cr Transport Characteristics of *Neyraudia reynaudiana* Under Cr(VI) Stress

**DOI:** 10.3390/plants15213423

**Published:** 2026-11-03

**Authors:** Fuyao Chen, Shaoliang Zhang, Huping Hou, Shi An, Min Tan

**Affiliations:** 1 School of Ecological Environment, Xuzhou Vocational College of Bioengineering, Xuzhou 221006, China; chenfuyao@cumt.edu.cn; 2 Research Center for Mine Ecological Restoration, Ministry of Education, China University of Mining and Technology, Xuzhou 221116, China; 3 School of Public Policy & Management, China University of Mining and Technology, Xuzhou 221116, China; hphou@163.com (H.H.);; 4 School of Public Policy & Management, Jiangsu Open University, Nanjing 210036, China; anshi@jsou.edu.cn

**Keywords:** antioxidant enzymes, Cr(VI) tolerance, contaminated soil, phytoremediation, translocation factor

## Abstract

Hexavalent chromium Cr(VI) contamination poses a significant risk to soil ecosystems because of its high mobility and toxicity. Identifying plant species capable of surviving under Cr stress and translocating Cr to harvestable aboveground tissues is a key step in developing phytoremediation strategies. Chromium (Cr) contamination, particularly hexavalent chromium Cr(VI), poses a substantial risk to soil ecosystems. This study evaluated the tolerance, Cr distribution, and antioxidant responses of *Neyraudia reynaudiana* grown in soil amended with K_2_Cr_2_O_7_. Seedlings were exposed to five nominal Cr treatments (200, 400, 600, 800, and 1000 mg kg^−1^) and sampled at 30, 60, 90, 120, and 150 days. Total Cr concentrations were determined in soil, roots, and shoots; translocation factor (TF) and shoot bioconcentration factor (BCF) were calculated. The activities of superoxide dismutase (SOD), peroxidase (POD), catalase (CAT), ascorbate peroxidase (APX), and glutathione reductase (GR) were also measured. *N. reynaudiana* remained viable throughout the 150 d exposure period, including under the highest nominal Cr treatment. At 150 d, TF exceeded 1.0 in the Cr400–Cr1000 treatments and reached a maximum of 1.50 in the Cr600 treatment, indicating substantial root-to-shoot Cr translocation under these conditions. In contrast, shoot BCF values remained below 0.30 (0.20–0.29 at 150 d), indicating limited concentration-based Cr enrichment in aboveground tissues. Soil total Cr concentrations declined during cultivation; however, biomass-based Cr mass balance is required to quantify net plant-associated Cr removal. The activities of SOD, POD, CAT, APX, and GR showed distinct Cr-concentration- and growth-stage-dependent responses, with the overall antioxidant response generally strongest at intermediate Cr concentrations and attenuated under the highest treatment. These findings indicate that *N. reynaudiana* has tolerance to Cr(VI) stress and the capacity for root-to-shoot Cr translocation in a controlled pot experiment. Its practical phytoremediation efficiency requires further evaluation using biomass-based Cr accumulation, Cr speciation, and field validation. By characterizing the Cr tolerance and translocation responses of a native slope-restoration species, this study provides a physiological basis for evaluating *N. reynaudiana* as a candidate plant for integrated stabilization and phytoextraction-oriented remediation strategies at Cr-contaminated sites. It also identifies the key evidence needed to translate pot-scale responses into reliable site-specific remediation assessments.

## 1. Introduction

Chromium (Cr) contamination is a widespread environmental problem associated with mining, metallurgy, electroplating, leather processing, and other industrial activities [1,2]. Cr occurs mainly as trivalent chromium (Cr(III)) and hexavalent chromium (Cr(VI)), but Cr(VI) is generally more mobile, bioavailable, and toxic [3,4,5,6]. Its effects on plants depend not only on the total Cr concentration but also on Cr speciation, soil properties, and bioavailability. Therefore, the environmental relevance of an experimental Cr treatment should be evaluated by considering both the nominal Cr addition and the measured Cr fraction available to plants [7,8].

Previous studies have shown that plant responses to Cr vary greatly among species. Cr-tolerant or Cr-accumulating plants such as *Spartina argentinensis*, *Prosopis laevigata*, and *Pteridium aquilinum* have been investigated for their ability to survive Cr exposure and accumulate or redistribute Cr in their tissues [9,10,11,12]. These studies indicate that Cr tolerance, tissue accumulation, and root-to-shoot translocation are related but distinct traits. Some species retain most Cr in roots, whereas others transfer a greater proportion to shoots; however, high tissue concentration does not necessarily indicate high total Cr removal because total accumulation also depends on plant biomass [13,14,15]. At high external Cr concentrations, uptake and translocation may become non-linear because of root retention, transport limitation, toxicity, or changes in soil Cr availability [16,17,18,19].

Cr stress is also associated with oxidative imbalance. Studies of different plant species have reported that the activities of superoxide dismutase (SOD), peroxidase (POD), catalase (CAT), ascorbate peroxidase (APX), and glutathione reductase (GR) may increase under low or moderate stress but decline when stress exceeds the plant’s compensatory capacity [20,21]. These enzymes can therefore provide physiological evidence of stress responses, although enzyme activity alone cannot establish the specific molecular mechanisms of Cr uptake, translocation, chelation, or vacuolar sequestration. In phytoremediation research, candidate plants should consequently be evaluated using multiple criteria, including survival, biomass production, root-to-shoot Cr distribution, biomass-based Cr accumulation, soil Cr behavior, and the feasibility of harvesting contaminated biomass [22,23].

*Neyraudia reynaudiana* is a perennial grass native to southern China and is widely used in slope restoration because of its developed root system and adaptability to drought, poor soils, and other challenging conditions [24]. Previous work has also examined its potential for managing Pb-contaminated soil [25]. However, Pb and Cr(VI) differ in chemical speciation, mobility, uptake pathways, and toxicity, so Pb-related evidence cannot be directly extrapolated to Cr(VI). Compared with the growing literature on other Cr-tolerant or Cr-accumulating plants, the responses of *N. reynaudiana* to Cr(VI) across a broad concentration gradient and successive growth stages remain poorly characterized. In particular, the relationships among plant survival, soil Cr change, root-to-shoot Cr distribution, and antioxidant enzyme activity require further clarification.

Therefore, this study used a controlled pot experiment to evaluate the tolerance, Cr distribution, and antioxidant responses of *N. reynaudiana* under K_2_Cr_2_O_7_ stress. We tested three hypotheses: (1) *N. reynaudiana* can maintain survival and growth under high Cr stress; (2) Cr is translocated from roots to shoots during plant development, but tissue Cr concentrations and translocation may show non-linear or saturating responses at higher external Cr levels; and (3) SOD, POD, CAT, APX, and GR activities vary with Cr concentration and growth stage and are associated with the physiological tolerance response. Seedlings were exposed to five nominal Cr treatments and sampled at five growth stages. Total Cr concentrations in soil, roots, and shoots were determined, translocation and bioconcentration indices were calculated, and the activities of the five antioxidant enzymes were measured. This study provides a basis for evaluating whether *N. reynaudiana* is a candidate species for integrated slope stabilization and Cr phytoremediation, while identifying the additional biomass-based and field evidence required for practical application.

## 2. Results

### 2.1. Change of Cr in Soil

**Figure 1:**
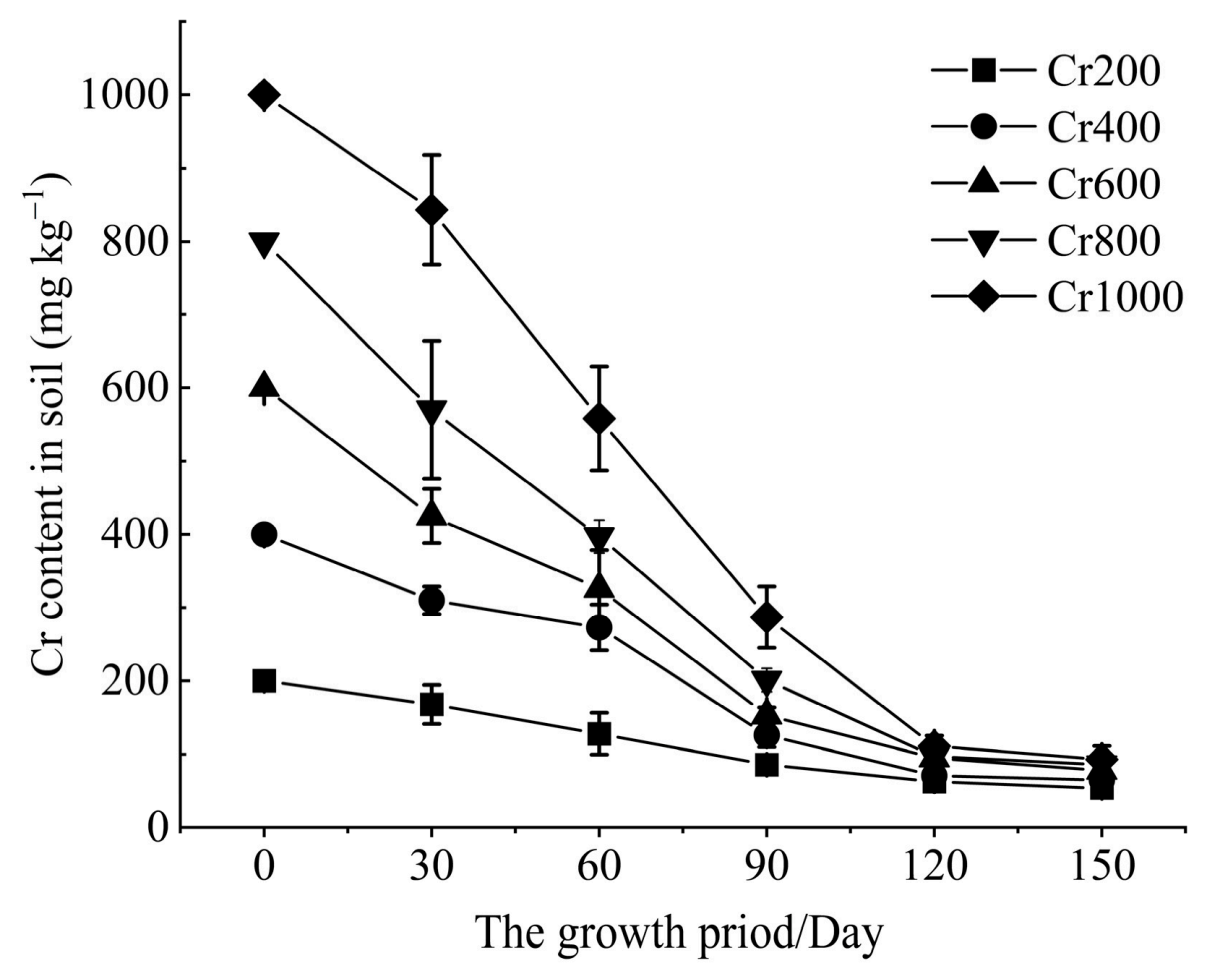
Changes in Cr content in soil during each growth period. Results are expressed as means ± SD (standard deviation). N = 5.

During plant growth under different concentrations of Cr stress, the Cr content in the soil varies (Figure 1). In the low-concentration groups (Cr200 and Cr400), the Cr content in the soil reaches its maximum at the YP stage and then gradually decreases to the lowest value at the AP stage. This indicates that as the plants grow, the Cr content in the soil gradually decreases. However, in the medium- and high-concentration groups, this change is more significant. In the Cr600 group, the Cr content in the soil was measured at 425 mg kg^−1^ at the YP stage and 326 mg kg^−1^ at the GP stage. Then, as the plants grew, the soil Cr content was measured as 153 mg kg^−1^ at the 90-day growth stage. In the later stage of plant growth, the Cr content in the soil decreased to 95 mg kg^−1^ at the OP stage and 78 mg kg^−1^ at the AP stage. Under high-concentration Cr stress, the Cr content in the soil changed more significantly within a shorter growth period.

The pollution rehabilitation index (PRI) values for *N. reynaudiana* at different Cr treatments and growth stages are presented in Table 1. Across all treatments, PRI generally increased with plant growth time. The PRI values ranged from 0.16 to 0.29 at the young plant stage (30 days), 0.32 to 0.50 at the growth stage (60 days), 0.57 to 0.75 at the mature stage (90 days), 0.69 to 0.89 at the old plant stage (120 days), and 0.73 to 0.91 at the apoptosis stage (150 days). At 150 days, the PRI increased with the nominal Cr treatment level, with values of 0.73, 0.84, 0.87, 0.89, and 0.91 for the Cr200, Cr400, Cr600, Cr800, and Cr1000 treatments, respectively. The highest PRI value was recorded in the Cr1000 treatment at 150 days.

### 2.2. Change in Cr Content in Roots

**Figure 2:**
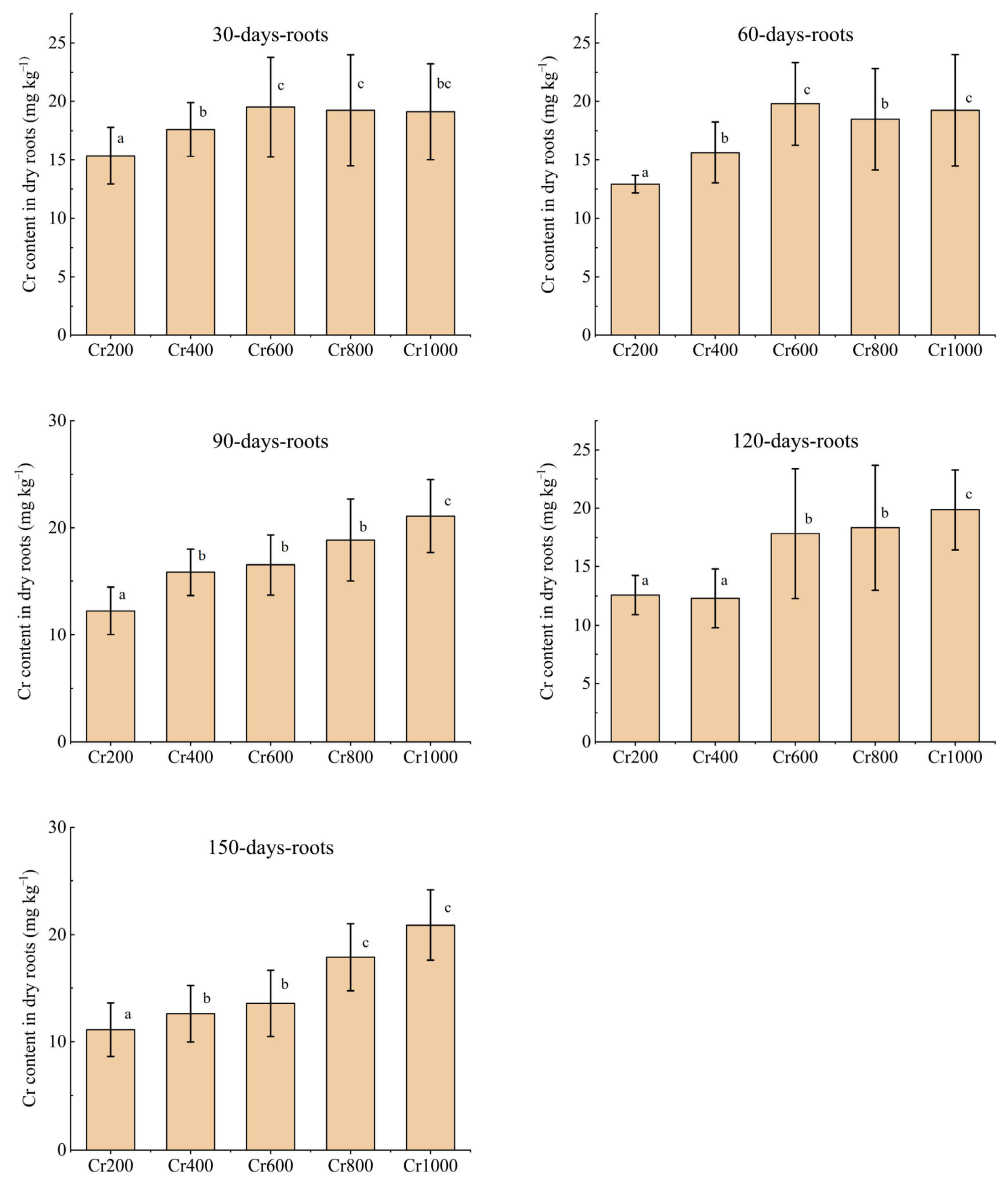
Change in Cr content in dry roots (mg kg^−1^). Results are expressed as means ± SD (N = 5). Different lowercase letters indicate significant differences among Cr concentration treatments, as determined by one-way ANOVA followed by Duncan’s multiple range test (*p* < 0.05).

As the first point of contact between plants and the external environment, the root system is the part that can most directly reflect the impact of heavy metals on plants. Therefore, the Cr concentration in the root system was determined at different growth periods and under different Cr stress concentrations. The results in Figure 2 show that under low-concentration stress (Cr200 and Cr400 groups), the Cr content in the plant root system gradually decreased with plant growth. The Cr concentration in the root system of YP stage was the highest, and that at the AP stage was the lowest. However, as the Cr content added to the soil increased, the pattern of Cr content in the root system changed. In the Cr600 group, the Cr content in the root system was in a disordered state. The minimum value was still recorded at the AP stage, with a Cr content of 13.57 mg kg^−1^ in the root system, while the maximum value was recorded at the GP stage, reaching 19.80 mg kg^−1^.

In contrast with the trend in the soil, the changes in Cr content in the root system of the high-concentration groups were no longer the same. The Cr800 group showed a gradually decreasing trend similar to that of the low-concentration groups, but the overall change was smaller. In the Cr1000 group, the Cr content in the root system of *N. reynaudiana* reached its peak at the MP period at 21.08 mg kg^−1^. From the YP stage to the MP stahe, it gradually increased, reached its peak, and then gradually decreased.

As the first step of the connection between soil heavy metals and plants, the root system will immediately experience damage to its cells when plants are exposed to heavy metals. The root system is an important organ for plants to absorb water and mineral elements, so the absorption factor can directly reflect the tolerance of the plant’s root system to soil heavy metals. Tolerance is the primary ability of plants used for phytoremediation. It can also reflect the correlation between the root system and the content of soil heavy metals. Although the amount of Cr added in various treatment groups is different, regardless of the change in the added Cr content, with the extension of the plant’s growth time, the *AF* of Cr by *N. reynaudiana* shows an increasing trend over time in Table 2. The longer the growth time, the higher the absorption rate of *N. reynaudiana*. Among them, in the Cr1000 group, when it grows to 150 days, the absorption rate of Cr by *N. reynaudiana* sinensis reaches 22.44%. By comparing the peak values of the transfer rate under various stress concentrations, it is found that as the content of the added exogenous Cr increases, the absorption rate of Cr by *N. reynaudiana* gradually increases, indicating that within the pollution concentration range designed in this experiment, *N. reynaudiana* can continuously tolerate the stress pressure of Cr and continuously transfer Cr from the soil into the root system.

### 2.3. Change in Cr Content in Shoots

**Figure 3:**
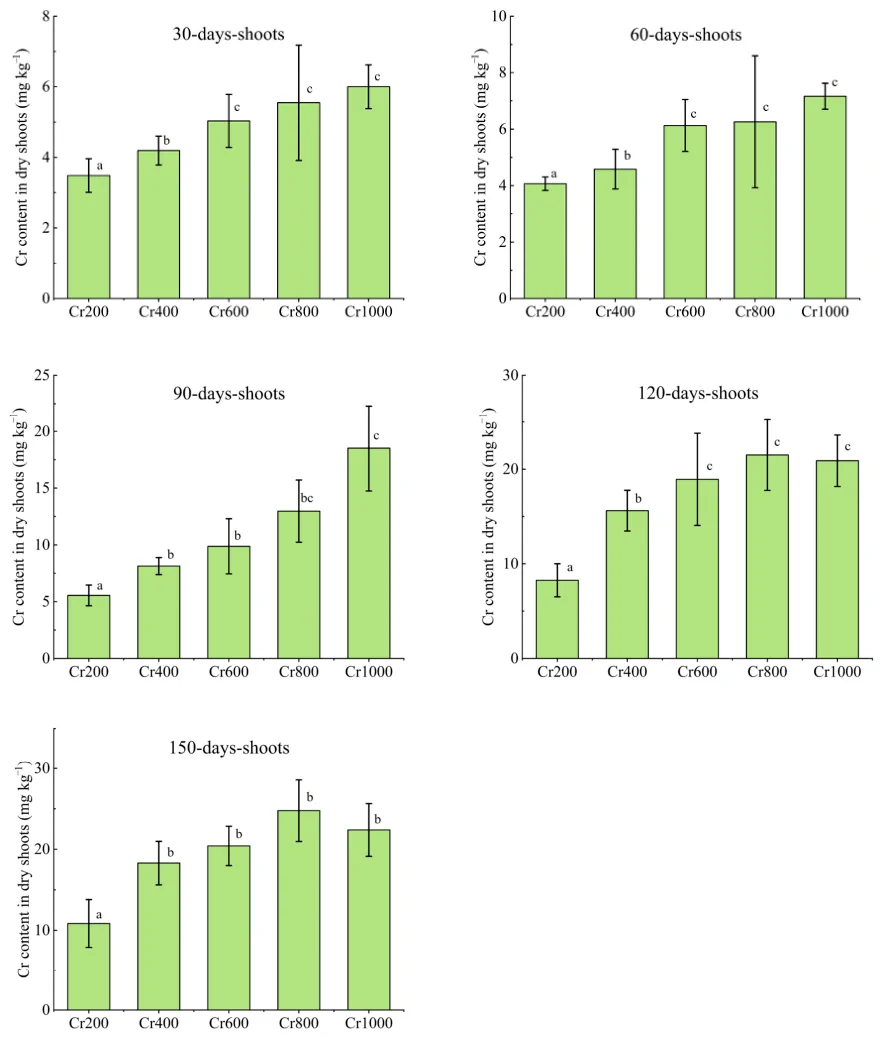
Change in Cr content in dry shoot (mg kg^−1^). Results are expressed as means ± SD (N = 5). Different lowercase letters indicate significant differences among Cr concentration treatments, as determined by one-way ANOVA followed by Duncan’s multiple range test (*p* < 0.05).

In order to achieve the goals of reducing heavy metal pollution concentration, restoring the soil’s practical functions, and minimizing the risk of pollution spread, the most fundamental approach is to transfer the heavy metals in the soil to the above-ground parts and then harvest the plants. Therefore, the content of Cr in the above-ground parts, especially in the leaves, can be an important factor determining whether the plants can be used as remediation plants. Therefore, the content of Cr in the leaves was measured, and the results are shown in Figure 3. In the medium- and low-concentration groups (Cr200, Cr400, and Cr600), as the plant growth time increased, the Cr content in the plant leaves gradually increased and reached its peak after 150 days of plant growth. However, as the Cr content added to the soil increased, the variation pattern of Cr content in the leaves showed differences. In the high-concentration (Cr800 and Cr1000) groups, the Cr content in the plant leaves was lower at the YP and GP stages, but as the plant grew, the Cr content rapidly increased after reaching the MP. In the Cr800 group, the Cr content in the leaves was only 6.26 mg kg^−1^ at the GP stage after 60 days of growth, but it would rapidly increase to 12.97 mg kg^−1^at the MP period of 90 days, and reach 24.77 mg kg^−1^ at the AP stage. In the Cr1000 group, similar changes occurred, with significant changes in Cr content in the leaves from the GP to MP stages.

The translocation factor (TF) reflects the transfer process of Cr within the plant. A higher TF indicates a greater accumulation of Cr in the aboveground parts of the plant, while the accumulation of Cr in the root system is relatively lower. For the remediation of heavy metals in soil, it is more desirable for the target plant to transfer as much of the heavy metals in the soil into its above-ground parts for accumulation and storage as possible. Therefore, we analyzed and discussed the translocation factor of *N. reynaudiana,* and the results are shown in Table 3.

Due to the different amounts of Cr added in each group, the translocation factors themselves vary. However, regardless of the variation in the added amount, the translocation factor in *N. reynaudiana* shows a gradually increasing trend as the plant growth time extends. The translocation factor reaches its maximum value at the AP stage. By comparing the peak values of the translocation factor under different stress concentrations, it is found that as the added external Cr content gradually increases, the translocation factor of *N. reynaudiana* increases first and then decreases. When the concentration increases to 600 mg kg^−1^, the translocation factor reaches its peak at 150.47%, and then begins to gradually decrease. This indicates that when the concentration exceeds 600 mg kg^−1^, the damage to *N. reynaudiana* caused by Cr, especially during the transfer process, begins to become irreversible.

The bioconcentration factor reflects the ability of the aboveground parts to accumulate heavy metals from the soil. The ultimate goal of phytoremediation is to achieve the transfer of heavy metals in the soil and change their storage locations, concentrating Cr in the aboveground parts of plants for easy removal and subsequent soil reuse. A higher BCF indicates a greater accumulation of Cr in the aboveground parts of *N. reynaudiana*, and correspondingly, a lower Cr content in the soil. Therefore, we calculated and analyzed the enrichment rate of Cr in *N. reynaudiana* at different growth stages, and the results are presented in Table 4.

Due to the different amounts of Cr added in each group, the bioconcentration factor varies. As the growth period of the plants extends, the BCF of *N. reynaudiana* shows a gradually increasing trend. As the plants grow, the BCF reaches the maximum value during the AP period. By comparing the peak enrichment rates under different stress concentrations, we found that as the content of exogenously added Cr increases, the BCF of *N. reynaudiana* for Cr shows a trend of first increasing and then decreasing. The BCF reaches its peak in the group with Cr600 and then decreases. This indicates that after the soil concentration exceeds 800 mg kg^−1^, the BCF of *N. reynaudiana* is affected.

### 2.4. Changes in Redox Enzyme Activity

Under Cr stress, the changes in the activities of the antioxidant enzyme system (SOD, POD, CAT, APX, and GR) in *N. reynaudiana* reveal a complex mechanism where the physiological responses of plants are regulated by the dual factors of soil heavy metal concentration and plant growth stage. This dynamic response process is the core physiological basis for *N. reynaudiana*’s tolerance to Cr pollution and its remediation.

**Figure 4:**
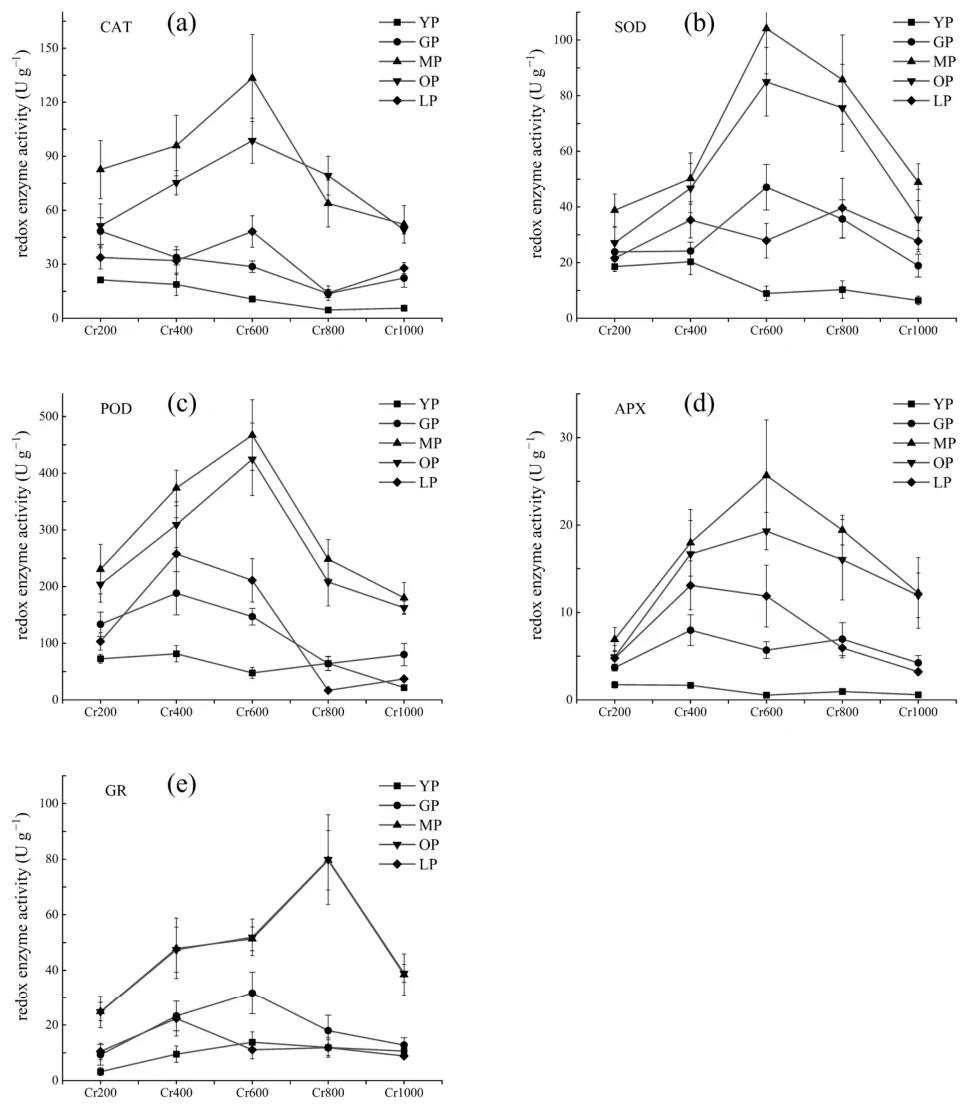
Changes in redox enzyme activity (U g ^−1^). Results are expressed as means ± SD, N = 5. (**a**) SOD, superoxide dismutase; (**b**) POD, peroxidase; (**c**) CAT, catalase; (**d**) APX, ascorbate peroxidase; and (**e**) GR, glutathione reductase. Cr0 denotes the control treatment without exogenous Cr(VI), whereas Cr200, Cr400, Cr600, Cr800, and Cr1000 denote the soil treatments with target Cr(VI) concentrations of 200, 400, 600, 800, and 1000 mg kg^−1^, respectively. YP, GP, MP, OP, and AP represent the young period (30 d), growth period (60 d), mature period (90 d), old period (120 d), and apoptosis period (150 d), respectively. Enzyme activities are expressed as U g^−1^. Data are presented as means ± standard deviation (SD), with five biological replicates per treatment group (N = 5).

As the growth stage progresses from the YP to the AP, the soil Cr stress concentration corresponding to the peak activity of various enzymes shows a regular shift. Plants in the middle and late growth stages (MP and OP) can tolerate and respond to medium and high concentrations of Cr stress (concentrations greater than 600 mg kg^−1^), while in the early or late growth stages, the activity peaks often occur in the low-concentration group. In the early growth stage, the antioxidant defense system tends to be rapidly activated by lower-concentration stress to cope with initial oxidative damage (the early response of CAT in Figure 4a); as the plant develops and biomass accumulates, its tolerance ability increases, and it can maintain stronger activity expression at higher stress concentrations to support the remediation function; in the late growth stage, the decline of some enzyme activity peaks may be related to resource redistribution and physiological function adjustment.

The Cr concentration corresponding to the peak SOD activity in Figure 4b gradually increases from Cr400 in the YP stage to Cr800 in the AP stage; CAT and APX are sensitive to low concentrations in the early stage, both shift to Cr600 in the middle stage, and APX returns to Cr400 in the late stage. In particular, the response pattern of GR shows a significant lag, with its activity peak stably occurring at higher stress concentrations throughout the growth period, which forms a temporal functional complementarity with other enzymes.

At the same time, within the same growth stage, the sensitivity of different enzymes to Cr concentration differs significantly, forming a well-structured and functionally coordinated defense network. In the early stage of stress, enzymes such as CAT respond first; as the concentration increases, core enzymes such as SOD, POD (in Figure 4c), and APX (in Figure 4d) are widely induced; and under high and continuous stress, enzymes such as GR play a key role in continuous detoxification (in Figure 4e). This division of labor enables the antioxidant system to flexibly adapt to different intensities of environmental pressure.

The changes in the antioxidant enzyme activities of *N. reynaudiana* are not static responses to a single environmental factor, but rather the dynamic result of the interaction between the growth and development program and environmental stress signals (Table 5). This growth stage-based plasticity regulation strategy enables *N. reynaudiana* to optimize its physiological resource allocation and establish effective detoxification and tolerance mechanisms in different growth stages and pollution levels, providing a key physiological perspective for understanding the adaptability and remediation potential of plants to heavy metals.

The response of enzyme activity to Cr concentration shows a clear concentration-dependent dynamic characteristic. In the medium and low-concentration groups, the average enzyme activity increased successively and reached a peak (422.57) in the Cr600 group, indicating that moderate and low concentrations of Cr may activate the plant’s antioxidant defense system and induce the upregulation of enzyme activity to cope with oxidative stress. However, as the Cr concentration continued to increase, the enzyme activity significantly declined, reflecting that the high concentration of Cr had exceeded the physiological tolerance range of the plant, resulting in the inhibition of the enzyme system function. The results of one-way analysis of variance and post hoc multiple comparisons showed significant differences between several groups, further indicating that different Cr concentration treatments had a significant impact on enzyme activity, especially under high-concentration conditions, where the enzyme activity was significantly inhibited.

**Figure 5:**
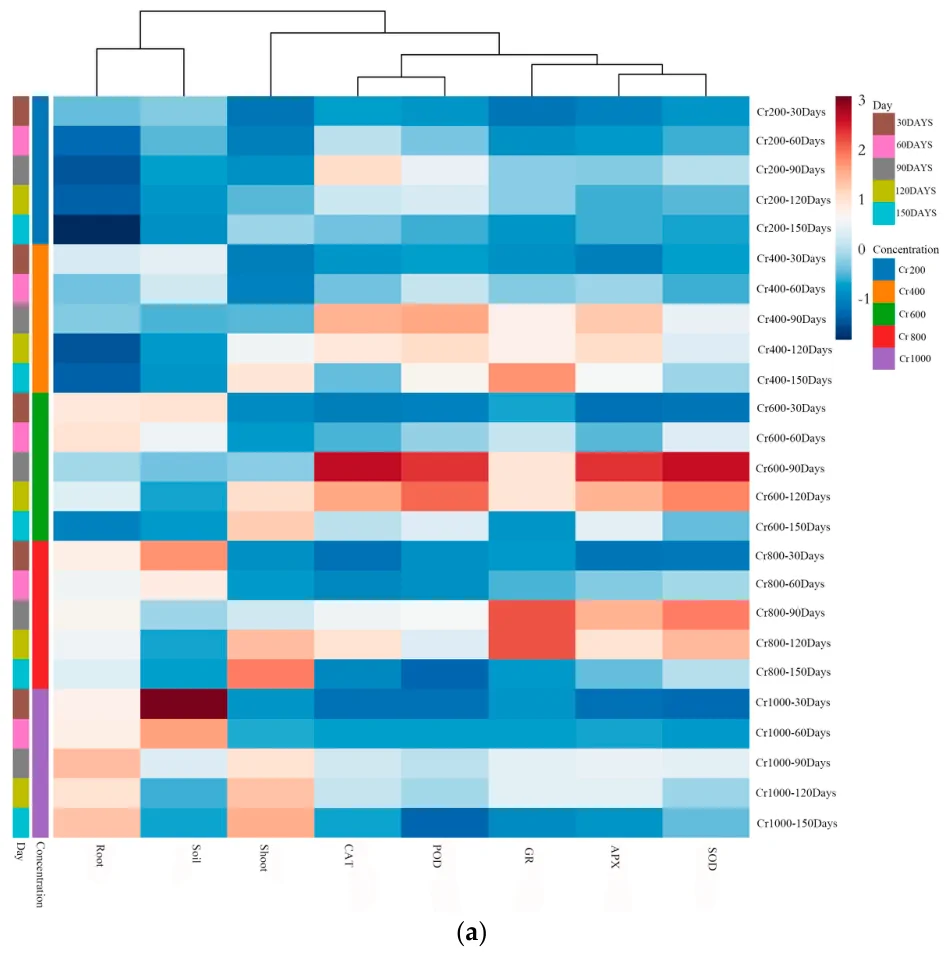

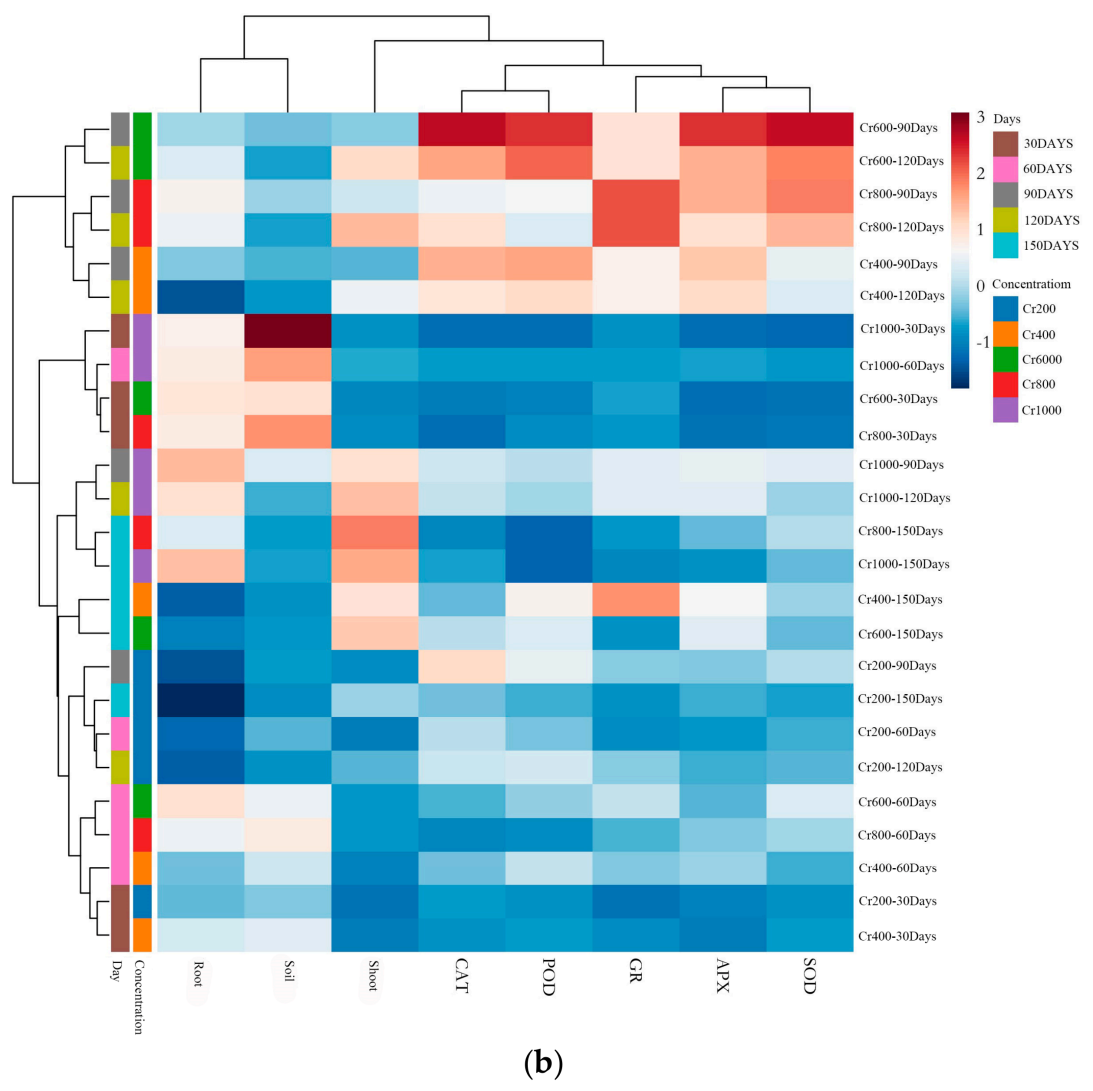
Heatmap: (**a**) the relationship between the activity of l antioxidant enzymes and Cr concentration; and (**b**) the relationship between antioxidant enzyme activity and growth time.

The heatmaps in Figure 5 present the association patterns among five antioxidant enzymes (CAT, POD, GR, APX, and SOD), five growth time points (30–150 days), and five Cr(VI) concentration gradients (Cr200-Cr1000) in roots, shoots, and soil. The color scale ranges from −1 to 3, with red indicating values above the control level and blue indicating values below the control level; the color-coded side bars denote growth duration and Cr concentration groupings, respectively. The heatmap in Figure 5a primarily reveals the concentration-dependent effects on antioxidant enzyme activities along the Cr gradient, whereas the right heatmap delineates the temporal dynamics of enzyme activities across the growth period.

Figure 5a illustrates the relationship between antioxidant enzyme activities and Cr concentration. The low-concentration groups (Cr200 and Cr400) are predominantly blue, indicating that antioxidant enzyme activities remain at relatively low levels and *N. reynaudiana* has not yet been subjected to pronounced oxidative stress. As the Cr concentration increases to Cr600 and Cr800, the activities of all five enzymes (CAT, POD, GR, APX, and SOD) rise synchronously, with the heatmap displaying extensive deep-red regions, reflecting a coordinated activation of the plant’s antioxidant defense system in response to moderate-to-high Cr(VI) stress. Notably, the response pattern at Cr1000 does not follow a simple linear enhancement; instead, the activities of several enzymes exhibit a decline, suggesting that the extreme concentration may have exceeded the compensatory capacity of the antioxidant system, resulting in a certain degree of oxidative damage. Furthermore, Cr concentrations in the roots and shoots show a consistent high-value trend with enzyme activities under high Cr levels, whereas the soil Cr content displays relatively minimal variation, implying that soil Cr concentration is not the sole driver of enzymatic responses and that a more intimate coupling exists between internal Cr accumulation and antioxidant defense within *N. reynaudiana*.

The heatmap Figure 5b delineates the dynamic relationship between antioxidant enzyme activities and growth time. A prominent high-response cluster (Cr600-90d, Cr600-120d, Cr800-90d, and Cr800-120d) emerges in the upper portion of the heatmap, indicating that moderate-to-high Cr concentrations elicit the strongest antioxidant responses and shoot Cr translocation during the mid-growth stage (90–120 days). In contrast, the lower portion (Cr200-30d and Cr400-30d) exhibits distinctly low-response blue regions, suggesting that the antioxidant system remains insufficiently activated under low-concentration early-stage stress. Cr1000 displays intense red signals as early as 30–60 days, implying that extreme Cr concentrations can trigger robust defense reactions at an early stage; however, the subsequent responses become increasingly divergent, with declines in several enzyme activities that may be attributable to physiological deterioration under prolonged stress. At 150 days, the response patterns across all concentration groups tend to disperse, suggesting that *N. reynaudiana* in the late growth stage may reallocate antioxidant resources through adaptive regulation or that the superimposition of senescence and stress leads to a diminished defensive capacity.

## 3. Discussion

### 3.1. Tolerance of N. reynaudiana Under Cr Stress

The research showed that *N. reynaudiana* could tolerate high Cr stress. In the present study, plants remained viable throughout the 150-day experiment, including under the highest nominal Cr treatment of 1000 mg kg^−1^. This result supports the tolerance of *N. reynaudiana* under the tested conditions. It is consistent with studies of several Cr-tolerant grasses and perennial plants that maintain physiological activity under Cr exposure, but differs from the severe growth inhibition and oxidative injury reported for Cr-sensitive crops [26,27,28,29,30,31,32]. The persistence of *N. reynaudiana* across the treatment gradient is also relevant to its use in slope restoration, where sustained vegetation establishment may be as important as the amount of Cr accumulated in plant tissues.

The present response should nevertheless be distinguished from Cr hyperaccumulation. Compared with reported Cr-accumulating species, including *Spartina argentinensis*, *Prosopis laevigata*, and *Pteridium aquilinum*, *N. reynaudiana* showed relatively low shoot Cr enrichment [33,34,35,36]. Its response is therefore better described as Cr tolerance accompanied by regulated internal distribution, rather than as strong tissue enrichment. Previous work on *N. reynaudiana* in Pb-contaminated soil supports its value as a restoration species, but Pb and Cr(VI) differ in mobility, uptake behavior, and toxicity; therefore, the Pb results provide ecological context rather than direct evidence of Cr remediation [24].

### 3.2. Developmental Cr Distribution and High-Concentration Response

The Cr would be translocated from roots to shoots during plant development and this process might become non-linear at high external Cr concentrations. The results support this interpretation [37]. At 150 days, TF exceeded 1.0 in the Cr400–Cr1000 treatments and reached 1.50 in the Cr600 treatment, indicating that shoot Cr concentration exceeded root Cr concentration at the later stage. This stage-dependent redistribution is consistent with previous studies showing that metal partitioning can change with plant development as root uptake, xylem transport, tissue growth, and senescence change [38,39].

However, the shoot BCF remained below 0.30, with values of 0.20–0.29 at 150 days. Thus, root-to-shoot redistribution was not accompanied by strong concentration-based enrichment relative to soil. This combination of TF > 1 and BCF < 1 differs from the high-shoot-accumulation pattern of classical hyperaccumulators [40], but is compatible with tolerant non-hyperaccumulating plants [41,42]. The similar root and shoot Cr concentrations in the Cr800 and Cr1000 treatments further suggest that Cr uptake or translocation did not increase proportionally with external Cr [43]. Root retention, transport limitation, toxicity, and changes in soil Cr availability are possible explanations reported for high-Cr responses, although these processes were not directly distinguished here. Because TF and BCF are concentration-based indices, and because the present experiment does not provide a complete biomass-based Cr balance, the decrease in soil Cr concentration should not be interpreted as a direct measure of plant-mediated Cr removal.

### 3.3. Antioxidant Responses Under Cr Stress

The results showed that SOD, POD, CAT, APX, and GR responded to both Cr concentration and plant developmental stage. The present results support this hypothesis: enzyme activities varied among treatments and growth stages, and the overall response was generally stronger at intermediate Cr concentrations than at the highest treatment. This pattern is consistent with the biphasic response frequently reported for plants exposed to Cr or other heavy metals, in which moderate stress activates antioxidant defenses whereas excessive or prolonged stress reduces the capacity for enzymatic compensation [44,45].

The results also indicate that the five enzymes did not respond identically. SOD and POD are commonly regarded as early components of the enzymatic defense system because they participate in the initial control of reactive oxygen metabolism, whereas CAT and APX contribute to hydrogen peroxide removal through partially complementary pathways. GR is associated with maintenance of the glutathione redox pool and may support the continuity of the ascorbate–glutathione cycle under sustained stress. The stage- and concentration-dependent differences observed in the present study are therefore consistent with coordinated but non-uniform adjustment of the antioxidant system, rather than with a single enzyme determining Cr tolerance. This interpretation also explains why the response of *N. reynaudiana* is not identical to the pronounced late-stage antioxidant decline reported in some Cr-sensitive crops.

The antioxidant data should not, however, be used to infer unmeasured molecular processes. Increased or sustained enzyme activity may accompany Cr tolerance, but it does not directly demonstrate changes in ROS, glutathione or phytochelatin pools, Cr transporters, or vacuolar sequestration [46,47]. The present results therefore support an association between antioxidant regulation and the observed tolerance phenotype, while the molecular links between redox adjustment and Cr distribution remain to be tested.

Overall, the three hypotheses are supported at the level of the measured responses. *N. reynaudiana* remained viable under the tested Cr gradient; Cr distribution shifted toward shoots during later development, with limited shoot enrichment and a possible high-concentration constraint; and the five antioxidant enzymes showed concentration- and stage-dependent responses. These characteristics distinguish *N. reynaudiana* from classical Cr hyperaccumulators but may be relevant to contaminated slopes where vegetation establishment and partial contaminant management are both required [48]. Further evaluation should combine biomass-based Cr accumulation, soil Cr speciation and bioavailability, and field-scale pe.

## 4. Materials and Methods

### 4.1. Plant Materials

**Figure 6:**
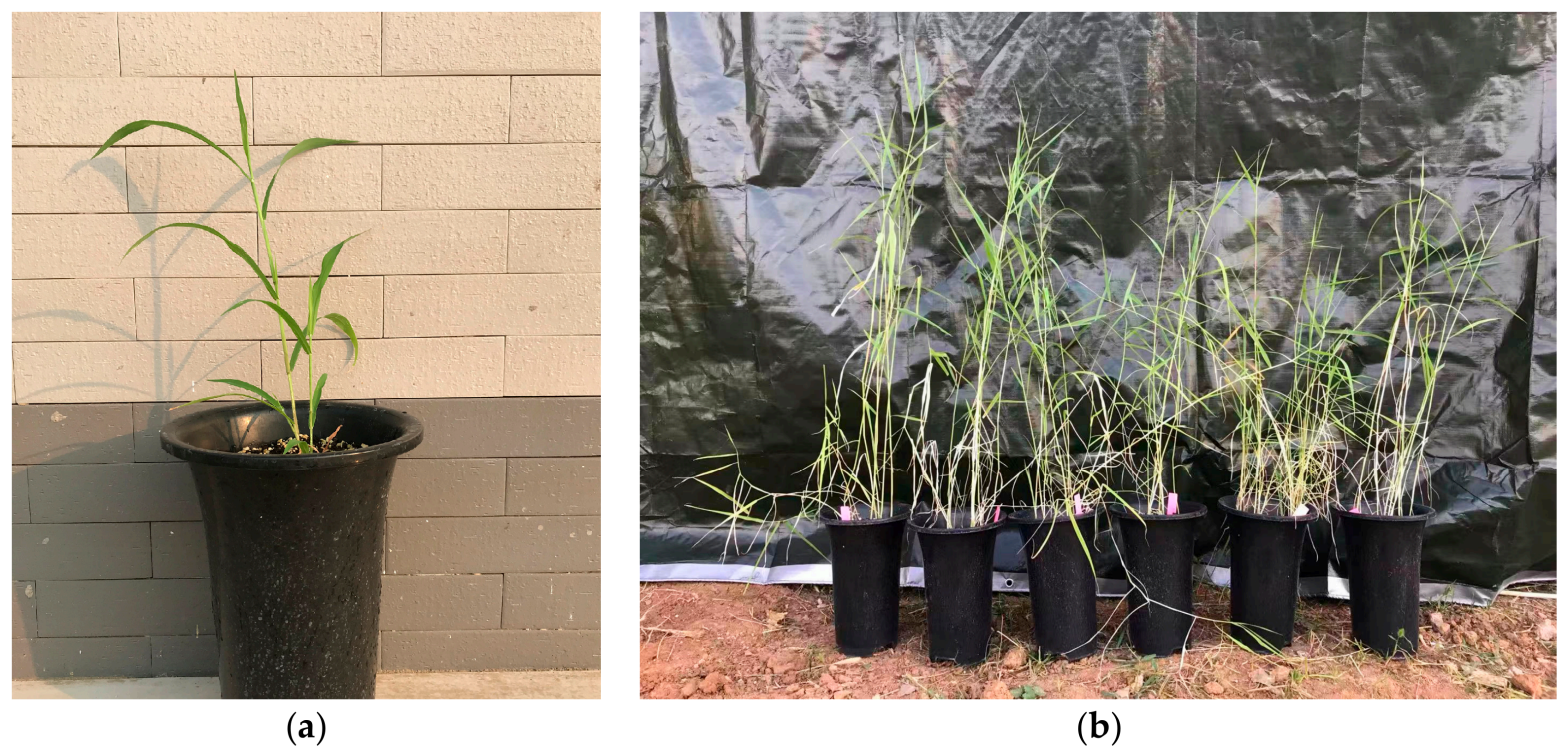
Images of *N. reynaudiana*: (**a**) before adding exogenous Cr in soil; and (**b**) after growing for 150 days. From left to right, the treatments range from Cr 200 to Cr 1000.

The seeds of *Neyraudia reynaudiana* were collected from Fuzhou, Fujian Province, China. Seeds were germinated on filter paper moistened with deionized water under dark conditions at 26 °C for 7 days. After the seedlings had grown to a height of 12 cm, they were transplanted into pots approximately 15 cm in height and 20 cm in diameter, with approximately 6 kg of soil added to each pot (Figure 6a). The soil was garden soil from Xuzhou, and potassium dichromate (K_2_Cr_2_O_7_) was mixed into the soil as the heavy metal source of Cr(VI). Five concentration gradients were set up with three replicates per gradient. The pH was maintained at 6.5, and K_2_Cr_2_O_7_ was mixed into the soil in appropriate proportions. After the soil and Cr(VI) were thoroughly mixed and allowed to equilibrate for two weeks, the *N. reynaudiana* seedlings were transplanted into the Cr(VI)-contaminated soil. The control group (Cr0) was set up with no Cr(VI) addition. According to the current definition of a Cr hyperaccumulator, the Cr content accumulated per kilogram of dry matter should exceed 1000 mg. Therefore, the highest concentration set in this experiment was 1000 mg kg^−1^. The concentration gradient design was also based on the national standard *Soil Environmental Quality—Risk Control Standard for Soil Contamination of Agricultural Land* (GB 15618-2018) [49]. Accordingly, five concentration gradients (200, 400, 600, 800, and 1000 mg kg^−1^) were established to span from just above the risk screening value to beyond the risk intervention value, thereby covering the full spectrum from moderate to severe Cr(VI) contamination representative of abandoned industrial sites in Xuzhou. This design enabled systematic evaluation of the tolerance threshold and accumulation capacity of *N. reynaudiana* across a realistic range of contamination intensities. 

The objective was to investigate whether *N. reynaudiana* can grow and accumulate Cr from the soil under high Cr(VI) stress. The specific concentrations are detailed in Table 6. During the cultivation period, the greenhouse was kept ventilated, with regular watering and supplementation of Hoagland [50] nutrient solution.

Soil samples from different depths (0–20 cm) were collected as experimental materials (Soil physical and chemical properties in Table 7). After natural air-drying and crushing, the soil samples were sieved for heavy metal content analysis. Shoot and root samples of *N. reynaudiana* were also collected. These samples were rinsed with ultrapure water, followed by three additional rinses, and then dried in an oven at 55 °C until constant weight. After crushing, the plant samples were sieved twice through a 100-mesh sieve, placed into sterile sealed bags, and numbered. Samples were collected five times throughout the growth cycle, corresponding to the young period (YP) at 30 days, the growth period (GP) at 60 days, the mature period (MP) at 90 days, the old period (OP) at 120 days, and the apoptosis period (AP) at 150 days. *N. reynaudiana* were shown in Figure 6b after 150 days.

The analytical procedures included the determination of total Cr in soil and plant tissues and the measurement of SOD, POD, CAT, APX, and GR activities in leaf extracts. Soil and plant samples were acid-digested for Cr analysis, whereas enzyme activities were determined spectrophotometrically using established assays.

### 4.2. Cr Quantification in Soil

Weigh the 0.20 g soil sample (through a 0.2 mm sieve) accurately into the polytetrafluoroethylene crucible. Before acid digestion, a small volume of deionized water was added to moisten the sample in the crucible. This step helped maintain slight moisture and ensured sufficient contact between the sample and the digestion acids. Add 5 mL HCl, heat it on the electric hot plate of the fume bonnet, and when it has evaporated to 2–3 mL, add 5 mL concentrated HNO_3_, 2 mL HF, 2 mL HClO_4_ successively, and heat it on the electric hot plate for about 1 h after covering. Shake the crucible frequently to ensure effective silicon fluxing. Open the lid and continue heating until thick white HClO_4_ smoke rises; cover the black organic carbide to decompose completely when the black organic matter is on the crucible; open the lid to expel the white smoke and vapors until the liquid is thick. The above decarburization process was repeated depending on the extent of decomposition. After cooling, the inner wall of the crucible was rinsed with 1% HNO_3_, and the resulting solution was filtered into a 25 mL colorimetric tube. The volume was adjusted to 25 mL, and the Cr concentration was finally determined with an atomic absorption spectrophotometer.

### 4.3. Cr Quantification in Different Tissues

Weigh 5 g of the plant sample, add 5 mL HNO_3_, and let it stand overnight. Then add 2 mL H_2_O_2_, followed by 4 mL H_2_O_2_. After shaking well, heat at 90 °C for 1 h. Then add 5 mL HNO_3_ and continue heating at 150 °C for one hour. After that, switch to heating at 120 °C until the sample is completely mineralized. Allow it to cool, and then adjust the volume to 25 mL by adding H_2_O. Shake well for the test. Cr content was quantified by inductively coupled plasma mass spectrometry (ICP-MS, Agilent 7500A, Santa Clara, CA, USA).

### 4.4. Quantification of Antioxidant Activity

Fresh leaf samples (0.20 g) were used to prepare crude enzyme extracts according to the instructions provided with commercial antioxidant enzyme assay kits from Sangon Biotech Co., Ltd. (Shanghai, China). The kits used in this study were Superoxide Dismutase (SOD) Activity Assay Kit (colorimetric method, Cat. No. D799593), Peroxidase (POD) Activity Assay Kit (colorimetric method, Cat. No. D799591), Catalase (CAT) Activity Assay Kit (UV-colorimetric method, Cat. No. D799595), Ascorbate Peroxidase (APX) Activity Assay Kit (UV-colorimetric method, Cat. No. D799461), and Glutathione Reductase (GR) Activity Assay Kit (micro method, Cat. No. D799262). The same crude enzyme extract was used for the determination of SOD, POD, CAT, APX, and GR activities. Absorbance values were determined spectrophotometrically at the corresponding wavelengths using a UV–Vis spectrophotometer.

SOD activity [51] was determined using the nitro blue tetrazolium (NBT) method. The crude enzyme extract was mixed with the corresponding reaction reagents and chromogenic reagents. After incubation for 20 min, the absorbance was measured at 560 nm.

POD activity [52,53] was determined using the guaiacol oxidation method. The reaction mixture contained 1.0 mL of crude enzyme extract, 1.0 mL of 1% guaiacol, and 1.0 mL of 0.18% hydrogen peroxide (H_2_O_2_). After incubation at 25 °C for 10 min, the reaction was terminated by adding 0.2 mL of 5% metaphosphoric acid, and the absorbance was measured at 470 nm.

CAT activity [52] was determined by monitoring the decomposition of H_2_O_2_ at 240 nm. The reaction mixture contained 0.2 mL of crude enzyme extract, 1.5 mL of phosphate buffer (pH 7.8), and 1.0 mL of deionized water. After equilibration at 25 °C, 0.3 mL of 0.1 mol L^−1^ H_2_O_2_ was added to initiate the reaction. Absorbance was recorded at 1 min intervals for 4 min.

APX activity [51] was determined in a 1.0 mL reaction mixture containing 50 mmol L^−1^ potassium phosphate buffer (K_2_HPO_4_–KH_2_PO_4_, pH 7.0), 0.5 mmol L^−1^ ascorbic acid, 0.1 mmol L^−1^ H_2_O_2_, and 0.5 mmol L^−1^ EDTA. The reaction was initiated by adding an appropriate aliquot of the crude enzyme extract. The decrease in absorbance associated with ascorbate oxidation was monitored at 290 nm at 25 °C.

GR activity [51] was determined in a 1.0 mL reaction mixture containing 100 mmol L^−1^ potassium phosphate buffer (K_2_HPO_4_–KH_2_PO_4_, pH 7.5), 2.5 mmol L^−1^ EDTA, 0.75 mmol L^−1^ 5,5′-dithiobis-(2-nitrobenzoic acid) (DTNB), and 1 mmol L^−1^ oxidized glutathione (GSSG). The reaction was initiated by adding NADPH to a final concentration of 0.1 mmol L^−1^ together with an appropriate aliquot of the crude enzyme extract. The absorbance was measured at 412 nm at 25 °C.

The activities of the five antioxidant enzymes were calculated according to the equations provided in the corresponding assay-kit protocols and expressed using the units specified for each assay. The same calculation procedures and units were used consistently in the text, tables, and figures.

### 4.5. Data Analysis

Cr concentrations in soil, roots, and shoots were expressed as mg kg^−1^ dry matter unless otherwise specified. The data are presented as means ± standard deviation (SD). All statistical analyses were performed using SPSS 25.0 (IBM Corp., Armonk, NY, USA).

Before conducting parametric statistical analyses, the data were assessed for normality and homogeneity of variance. Differences among the Cr treatment groups were evaluated using one-way analysis of variance (ANOVA). When significant differences were detected, Duncan’s multiple-range test was used for post hoc comparisons. Statistical significance was set at *p* < 0.05. Figures were prepared using Origin 2018 (OriginLab Corp., Northampton, MA, USA) and RStudio 2024.12. 

The following indices were calculated based on the measured Cr concentrations in soil and plant tissues:

$$AF=\frac{C_{\mathrm{root}}}{C_{\mathrm{soil}}}$$

where AF is the root absorption factor [54], $C_{\mathrm{root}}$ is the Cr concentration in roots, and $C_{\mathrm{soil}}$ is the Cr concentration in the corresponding soil sample.

$$TF=\frac{C_{\mathrm{shoot}}}{C_{\mathrm{root}}}$$

where TF is the translocation factor [55], $C_{\mathrm{shoot}}$ is the Cr concentration in shoots, and $C_{\mathrm{root}}$ is the Cr concentration in roots.

The bioconcentration factor [55] was calculated as:

$$BCF=\frac{C_{\mathrm{plant}\;\mathrm{tissue}}}{C_{\mathrm{soil}}}$$

where $C_{\mathrm{plant}\;\mathrm{tissue}}$ represents the Cr concentration in the plant tissue analyzed. The corresponding plant tissue is specified in the relevant table or figure.

The pollution rehabilitation index [55,56,57] was calculated as follows:

$$PRI=\frac{C_{s}-C_{e}}{C_{s}}$$

where $C_{s}$ is the initial Cr concentration in the soil, and *C*_e_ is the Cr concentration in the soil at the end of the cultivation period.

All concentration values used in these calculations were obtained from the corresponding measured soil or plant samples and were expressed on a consistent dry-mass basis.

rformance before practical phytoremediation efficiency is assigned.

## 5. Conclusions

Under the conditions of this controlled pot experiment, *Neyraudia reynaudiana* remained viable under a broad range of Cr(VI) stress levels and showed stage-dependent redistribution of Cr from roots to shoots. However, the limited Cr enrichment in shoots indicates that the species should not be regarded as a classical Cr hyperaccumulator. The concentration- and growth-stage-dependent responses of SOD, POD, CAT, APX, and GR further suggest that dynamic antioxidant regulation contributes to its physiological response to Cr stress.

Overall, *N. reynaudiana* shows potential as a candidate species for vegetation establishment and integrated management of Cr-contaminated slopes, but its practical phytoremediation efficiency cannot be determined from soil Cr changes or concentration-based indices alone. Future studies should combine biomass-based Cr mass balance, soil Cr speciation and bioavailability analyses, and multi-season field trials across different soil types. These investigations will help determine whether the tolerance, root-to-shoot Cr transfer, and antioxidant responses observed in this study can be translated into reliable phytostabilization or phytoextraction-oriented applications.

## Figures and Tables

**Table 1:** The PRI of Cr in each growth period of *N. reynaudiana*.

Number	Growth Period				
	YP 30 Days	GP 60 Days	MP 90 Days	OP 120 Days	AP 150 Days
Cr200	0.16 ± 0.01	0.36 ± 0.02	0.57 ± 0.03	0.69 ± 0.03	0.73 ± 0.03
Cr400	0.23 ± 0.02	0.32 ± 0.02	0.69 ± 0.03	0.16 ± 0.01	0.84 ± 0.09
Cr600	0.29 ± 0.01	0.16 ± 0.01	0.75 ± 0.02	0.84 ± 0.05	0.87 ± 0.07
Cr800	0.29 ± 0.01	0.5 ± 0.02	0.75 ± 0.05	0.88 ± 0.05	0.16 ± 0.01
Cr1000	0.16 ± 0.02	0.44 ± 0.04	0.16 ± 0.01	0.89 ± 0.04	0.91 ± 0.07

Results are expressed as means ± sd.

**Table 2:** The *AF* of Cr in each growth period of *N. reynaudiana*.

Number	Growth Period				
	YP	GP	MP	OP	AP
Cr200	0.09 ± 0.02	0.10 ± 0.02	0.14 ± 0.02	0.20 ± 0.03	0.21 ± 0.04
Cr400	0.06 ± 0.02	0.06 ± 0.01	0.13 ± 0.03	0.17 ± 0.01	0.19 ± 0.05
Cr600	0.05 ± 0.02	0.06 ± 0.01	0.11 ± 0.02	0.19 ± 0.02	0.17 ± 0.03
Cr800	0.03 ± 0.01	0.05 ± 0.02	0.09 ± 0.01	0.19 ± 0.03	0.21 ± 0.05
Cr1000	0.02 ± 0.01	0.03 ± 0.01	0.07 ± 0.02	0.18 ± 0.04	0.22 ± 0.06

Results are expressed as means ± sd.

**Table 3:** The *TF* of Cr at each growth stage of *N. reynaudiana*.

Number	Growth Period				
	YP	GP	MP	OP	AP
Cr200	0.23	0.31	0.45	0.66	0.97
Cr400	0.24	0.29	0.51	1.27	1.45
Cr600	0.26	0.31	0.60	1.06	1.50
Cr800	0.29	0.34	0.69	1.17	1.39
Cr1000	0.31	0.37	0.88	1.05	1.07

Results are expressed as means.

**Table 4:** The BCF of Cr in each growth period of *N. reynaudiana*.

Number	Growth Period				
	YP	GP	MP	OP	AP
Cr200	0.02	0.03	0.07	0.13	0.20
Cr400	0.01	0.02	0.08	0.22	0.28
Cr600	0.01	0.02	0.11	0.20	0.26
Cr800	0.01	0.02	0.08	0.22	0.29
Cr1000	0.01	0.01	0.13	0.19	0.24

Results are expressed as means.

**Table 5:** The comparative analysis of the REDOX activity under the Cr stress in *N. reynaudiana*.

	Cr0	Cr200	Cr400	Cr600	Cr800	Cr1000
Average	28.83	240.90	370.13	422.57	254.82	183.84
SD	13.84	107.20	177.88	296.60	204.63	125.29
F-value	3.10					
Cr0		−212.07	−341.31 *	−393.74 *	−225.99	−155.01
Cr200			−181.67	−13.92	57.07	−57.07
Cr400				115.31	186.29	−186.29
Cr600					238.80 *	−238.73 *
Cr800						−70.98

Data were analyzed by one-way ANOVA followed by Duncan’s multiple range test. * Indicates significant differences at the 0.05 level (*p* < 0.05).

**Table 6:** Addition and concentration of heavy metals in soil.

Number	Concentration/mg kg^−1^	K_2_Cr_2_O_7_/g Each Pot
Cr200	200	6.79
Cr400	400	13.58
Cr600	600	20.37
Cr800	800	27.16
Cr1000	1000	33.95

**Table 7:** Soil physical and chemical properties.

AK	AP	AN	SOC	pH	Cr
mg kg^−1^	mg kg^−1^	mg kg^−1^	%	-	mg kg^−1^
21.12	5.06	42.39	1.38	7.2	ND

## Data Availability

The data presented in this study are available upon request from the corresponding author.
